# ForamJ – A tool for the reproducible, semi-automated analysis of foraminifera micro computed tomography datasets

**DOI:** 10.1038/s41598-026-43276-3

**Published:** 2026-03-24

**Authors:** Jacob Trend, Fernando Alvarez Borges, Tali L. Babila

**Affiliations:** 1 School of Ocean and Earth Sciences, National Oceanographic Centre, University of Southampton, Southampton, SO14 3ZH UK; 2 Faculty of Engineering and Physical Sciences, University of Southampton, Southampton, SO17 1BJ UK; 3 Department of Earth, Environmental, and Planetary Sciences, Case Western Reserve University, Cleveland, 44106 USA

**Keywords:** Foraminifera, Micro computed tomography, Image analysis, Workflow development, Biological techniques, Computational biology and bioinformatics

## Abstract

The application of micro-computed tomography (µCT) to foraminiferal test analysis has opened new avenues for high-resolution, non-destructive 3D characterisation of internal and external morphological features. However, existing workflows are typically limited by low throughput, manual segmentation, and reliance on proprietary software, constraining dataset reproducibility and scalability. Here we present ForamJ, an open-source ImageJ plugin developed specifically for the semi-automated, reproducible analysis of foraminiferal µCT datasets. ForamJ streamlines key stages of image processing, including test segmentation, homogenous infill removal, chamber isolation, and morphological quantification, with outputs tailored to established micropaleontological metrics while providing a significant decrease in processing time versus existing methods. These include both calcite volume and chamber volumes, test surface area, inner and outer wall thickness and chamber centroids - parameters directly relevant to taxonomy, taphonomy, paleoecology, and biomineralisation processes. The plugin supports both single-sample and batch-processing workflows, enabling high-throughput analysis. Two use cases are presented: (1) the assessment of species-specific morphological variability in ancient cosmopolitan benthic foraminifera genus *Cibicidoides* spp. and (2) quantification of trends in between multiple species of modern planktonic foraminifera. Validation against manual segmentation of benthic foraminifera performed in commercial software ORS Dragonfly demonstrates ForamJ’s analytical fidelity, with a 1.30% difference in calcite volume between ForamJ and manually annotated tests, with a 0.9981% (± 0.001) segmentation accuracy and 0.97 (± 0.008) dice score with a median processing speed of 154 seconds per sample. By embedding foraminifera-specific metrics into an accessible, reproducible, and extensible framework, ForamJ provides a lightweight, dedicated digital toolset for advancing µCT-based test analysis in foraminiferal research.

**Supplementary Information:**

The online version contains supplementary material available at 10.1038/s41598-026-43276-3.

## Introduction

Micro-computed tomography (µCT) is a widely used 3D imaging technique across disciplines such as life sciences^1–3^, geosciences^4^, materials science^5^, and paleontology^6^. Its key strength lies in its ability to provide high-resolution, three-dimensional, non-destructive visualisation of internal structures from a single scan. This generates rich volumetric datasets, often necessitating the development of tailored image analysis pipelines to extract meaningful, quantitative information.

One promising application of µCT is for the study of foraminifera; unicellular, sub-millimetre-sized, shelled amoeboid protists. Foraminifera are a predominantly marine group, inhabiting sediments on the seafloor (benthic) and within the upper ocean’s water column (planktonic). Foraminifera produce multi-chambered calcium carbonate shells, or tests, whose morphology varies significantly between species and can reflect the climatic and environmental conditions in which calcification occurred^7,8^. Upon death, these tests are deposited and accumulated in seafloor marine sediments, where they can be preserved for millions of years, forming an extensive geologic archive for paleoenvironmental and paleoclimatic reconstructions^9^^,^^10^. µCT enables detailed imaging of both external test morphology^11–14^ and internal chamber architecture^11,15–19^, providing insights into taxonomy, taphonomy, growth patterns, and environmental responses. However, challenges arise due to intra-species morphological variability, the need for statistically robust sample sizes, and post-mortem alterations such as chamber wall dissolution and infilling, which complicate the segmentation processing and therein interpretation of original versus postmortem morphological features. These factors often necessitate manual input, reducing throughput and reproducibility. Therefore, to date, µCT imaging in foraminifera studies are generally limited in their test morphology parameterisation and sample size dataset. Thus, there is a need for image analysis tools to be optimised to handle large datasets and to accommodate for morphological variation between specimens. This must be paired with accurate, quantitative outputs, while minimising highly time-consuming and specialised imaging training required for manual segmentation.

Existing commercial image analysis software - such as ORS Dragonfly (Comet Technologies Canada Inc.), Avizo (Thermo Fisher Scientific Avizo), VG Studio (Volume Graphics GmbH) and IPSDK (IPSDK Explorer, Reactiv’IP) - are widely used for µCT segmentation, but are proprietary and to our knowledge, do not provide workflows specifically optimised for foraminiferal morphology, batch processing, and standardised chamber-level quantification. Several independent workflows for foraminiferal µCT analysis have been developed in the literature, including recent artificial intelligence (AI) based approaches using manually annotated datasets to train deep learning pipelines, automating test and chamber segmentation^20^. In parallel, continued advances in interactive and GUI-based frameworks for 3D biological imaging have lowered technical barriers for non-specialist users^21,22^. However, AI–based workflows typically still require manually annotated training data, model configuration, and computational resources, which can limit accessibility and reproducibility in foraminiferal studies. Individual studies have also began to address issues presented by foraminifera samples, such as chamber-to-chamber merging as a result of septal dissolution^23^, and the development of specific quantitative parameters such as chamber trochospirality^24^. However, an integrated workflow combining segmentation with downstream extraction of phenotypically relevant parameters for foraminiferal analysis is currently lacking.

To address this gap, we developed an open-source plugin for use within the ImageJ environment suite^25^, enabling a streamlined, reproducible and self-guided workflow for µCT analyses of foraminifera. While ImageJ supports a range of domain-specific plugins, for example, in bone biology^26^, neuroscience^27,28^ and ecology^29^. However, no such tool currently exists for foraminiferal research. Our plugin – ForamJ - aims to provide the foraminiferal research community with a dedicated, accessible solution for the quantitative analysis of µCT datasets.

### Approach

The primary goal of this plugin is to provide an accessible and reproducible image analysis workflow tailored to the foraminiferal research community. A significant barrier to wider adoption of µCT-based analysis in this field is the reliance on commercial software, which often requires access to high-performance imaging workstations and specialised training. Even when such infrastructure is available, method development can be time-consuming, user dependent and inconsistent across research groups. Furthermore, study of foraminifera often requires large sample sets to adequately characterise intra- and inter-population morphological variability high sample numbers and as such, a key aim was to make it scalable for larger datasets.

To address these limitations, the plugin was designed with the following core principles:

1. **Accessibility** – It must be intuitive and usable by researchers with beginner level image analysis experience.
2. **Portability** – It must be able to run on standard, non-specialist computing hardware.
3. **Open Source** – It must function entirely within the open-source ImageJ ecosystem.
4. **Reproducibility** – It must produce standardised, quantitative outputs in a consistent and easily interpretable format.
5. **High throughput** – The plugin must provide a significant improvement in processing speed, with potential for batch processing.

**Fig. 1:**
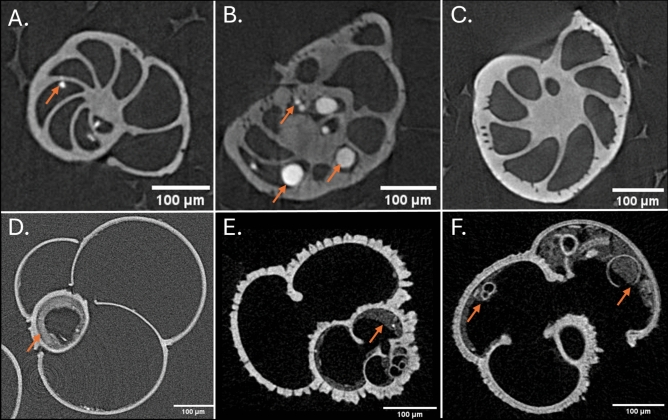
Sources of variability in µCT scans of foraminifera. Orange arrows highlight heterogeneous fine and coarse grain sediment infill within foraminifera chambers, illustrating differences in infill spatial density across specimens. Additional variation in image contrast is also evident between samples. Panels A–C show benthic species acquired during this study, while panels D–F show planktonic species accessed from the open repository provided by^30^.

To meet these criteria, we implemented a set of morphological measurements targeting both whole test and internal chamber architecture, based on established metrics described in the literature (Table 1). These measurements were selected to maximise biological relevance while ensuring they could be extracted reliably from typical µCT datasets, despite the presence of sediment infill, poor preservation and variable contrast between individuals. These factors may lead users to resist a one-size-fits-all approach to image segmentation (Fig. 1) or necessitate a complex - and often inaccessible to new users - machine learning or deep learning-based solution to account for this variability. During the development of ForamJ, it was noted that the specimens that were used for the development of this plugin, were relatively well preserved as illustrated in Figure 1.

**Table 1:** Table of parameters incorporated within ForamJ.

**Parameter**	**Unit**	**Description**	**Measurements reported in previous studies**
Calcite volume	µm^3^	Total volume of the test, excluding the chamber volumes	^11–14^
Test volume	µm^3^	Test volume including chamber volumes	^31^^,^^32^
Surface area	µm^2^	Surface area of filled calcite 3D volume	^33^
Internal chamber volume	µm^3^	Volume of internal chambers, summed	^11,15–18^
Chamber centroid	Coordinates, x y and z.	X-Y-Z coordinates of each chamber	^19^
Inner septa thickness	µm^3^	Average thickness of the inner septa	^34–36^
Outer test thickness	µm^3^	Average thickness of the outer test	^36–38^

### Installation and download

For local imagej/Fiji installation download the .ZIP file for your operating system from imagej.net/software/fiji/downloads, extract the folder (e.g., Fiji.app), and run the executable. Prior to running ForamJ from the ImageJ interface, the following plugins must also be installed: BoneJ^26^, 3D imageJ Suite^39^, IJ-plugins^40^ and Morphology^41^. For plugins, use Help > Update within Fiji to manage update sites or manually drag JAR/class files into the Fiji.app/plugins/ folder and restart Fiji. Following download of the .ijm file from the supplementary information of this article, it must be dropped into the plugins folder of the local Fiji installation and imageJ/Fiji restarted. ForamJ will be maintained on the corresponding authors GitHub, at the url: https://github.com/JTrendFiji/ForamJ. All method development was completed in Fiji ver 2.16.0.

### Methodological workflow

**Fig. 2:**
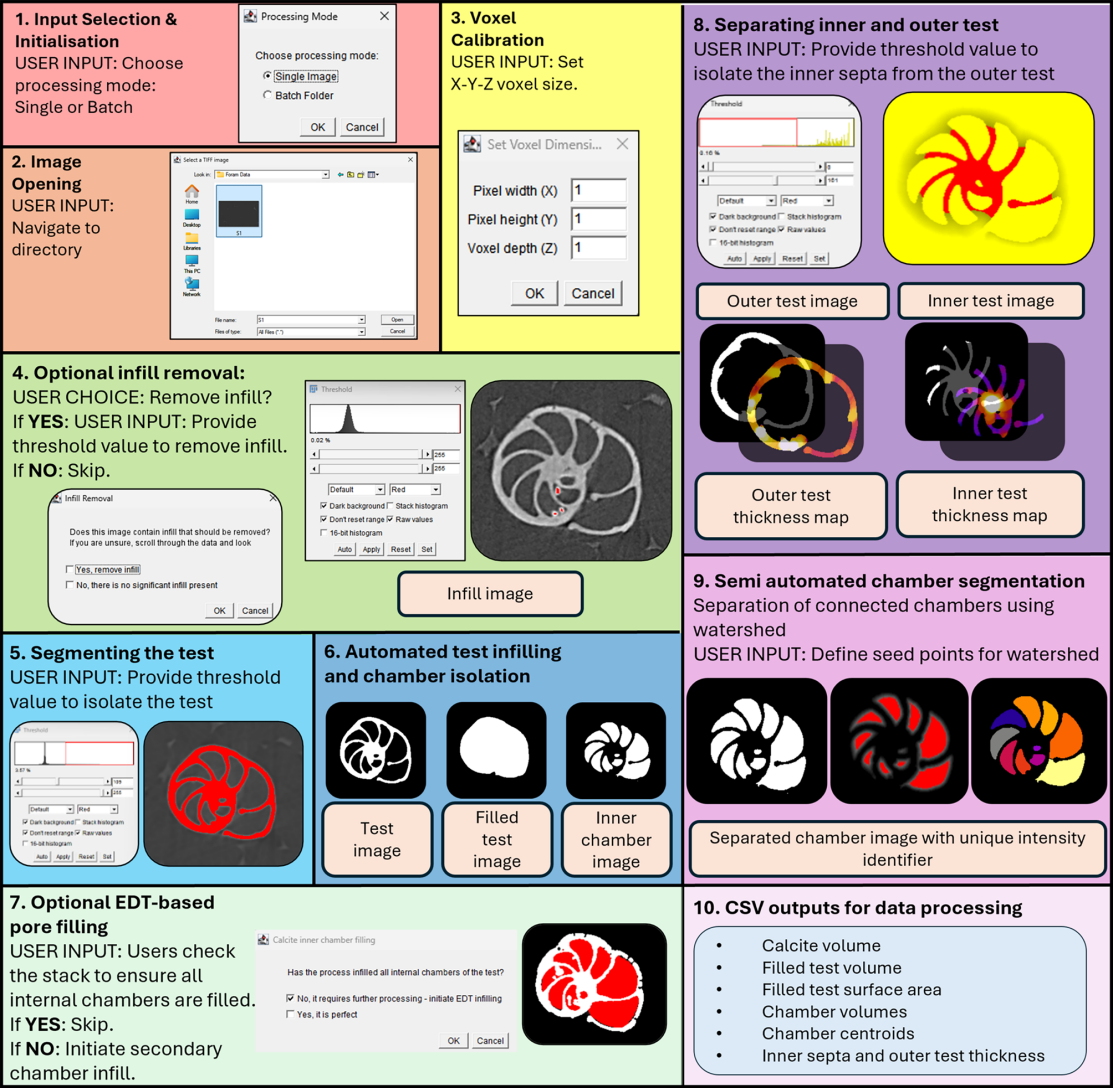
ForamJ workflow for segmentation and morphometric analysis of foraminiferal µCT datasets. The procedure begins with (1) input selection (single image or batch) and (2) file opening, followed by (3) voxel calibration to set X–Y–Z dimensions. (4) Optional infill removal allows users to exclude artificial material prior to analysis. (5) Thresholding isolates the test from background, leading to (6) automated chamber infilling and chamber mask generation. (7) An optional EDT-based pore filling step ensures internal chambers are completely closed. (8) Threshold-based separation of the inner septa from the outer test produces (9) thickness maps and enables semi-automated chamber segmentation using watershed with user-defined seed points. (10) The workflow outputs multiple CSV files containing quantitative descriptors including calcite volume, filled test volume, surface area, chamber volumes, centroids, chamber counts, and septal thickness.

Image stacks (of greyscale .tif or .tiff files) are processed using a custom ImageJ macro designed to support both Single Image and Batch modes. The processing workflow executed by this plugin is schematically presented in Fig. 2. The user then specifies whether to use Single Image model or Batch Mode (Fig. 2.1) before directing the plugin to the desired folder location. In Single Image mode, the user selects a single .tif file for manual processing . In Batch mode, all .tif or .tiff files within a selected directory are processed sequentially  . For both Single Image and Batch mode, ForamJ analyses one individual specimen per scan, akin to those produced by Searle-Barnes et al.^42^, whereby each field of view contains only a single specimen. Should the user scan multiple samples within a singular field of view, specimens should be separated first, into separate datasets, with one sample per tiff stack.

For each dataset, the macro creates two output directories: one for image outputs (Sample_Images) and one for tabulated results (Sample_Results), where Sample refers to the base filename. No known naming rules apply here, although we recommend avoiding characters that are invalid or problematic in file systems (such as /, \, :, *, ?, ", <, >, |). Voxel dimensions in the X, Y, and Z directions (µm) are specified by the user to ensure spatial scaling (Fig. 2.3). An optional infill removal step is included, in which users inspect the image stack and, if necessary, apply a manual threshold to generate an infill mask (Fig. 2.4). This mask is subtracted from the original dataset using image arithmetic, yielding a cleaned volume referred to as the Raw image.

Segmentation of the foraminiferal test begins by duplicating the Raw volume and applying a 3D Gaussian blur to reduce image noise, followed by thresholding defined by the user (Fig. 2.5). A "keep-largest-region" operation is used to eliminate non-target debris, and a morphological closing sequence (3D dilation, hole filling, erosion) generates a filled outer mask (FilledMask) representing the complete test morphology (Fig. 2.6). Here, the user is asked to confirm whether the test is completely filled. If yes, then the user moves onto the next step. If not - and some inner chambers are not infilled - the user should seect “no” to direct the use of a 3D Euclidean Distance Transform (EDT) to infill the inner chambers (Fig. 2.7). Specifically, a distance map is created from the filled calcite ROI, and the user then choses a threshold which seals off chambers which have remained open. This is followed by a second EDT which is overlaid onto the binary skeleton image, with the user once again selecting a threshold to infill the remaining test, ensuring that the threshold does not spill over the test outer shell, as to not artificially augment the test size. Once complete, this image is renamed to replace the previous filled outer mask. An approximation of surface area is computed from the FilledMask by identifying edge voxels and multiplying their count by the average voxel face area based on the user-defined voxel dimensions. This metric is exported as a .CSV.

To delineate septal and outer wall structures, a 3D Euclidean Distance Transform (EDT) is applied to the FilledMask. Users then threshold the EDT to isolate the septal region (Fig. 2.8), and the outer test and internal septa are exported as individual binary masks. A 3D thickness analysis (BoneJ plugin) is performed on each, yielding thickness maps and summary statistics.Subtracting the initial skeleton from the FilledMask produces a binary representation of the internal chamber volume (InnerChambers). For chamber segmentation, the InnerChambers volume is smoothed with a 3D Gaussian filter, and an EDT is applied. Users then generate seed regions via binary thresholding, ensuring chambers are spatially distinct. These seeds are labelled using 3D connected components analysis and passed to a 3D watershed algorithm, enabling segmentation of discrete chambers even when septal walls are partially degraded (Fig. 2.9). Each chamber’s centroid, volume, and average intensity are extracted and saved.

The plugin also computes total volumes for the SkeletonBinary, FilledMask, and InnerChambers masks by summing voxel counts and scaling by voxel volume. These values, along with all intermediate and final masks (e.g., distance maps, watershed labels, thickness maps), are saved as .tif images and .csv files for reproducibility and downstream analysis (Fig. 2.10). At each manual thresholding step, users are prompted to enter the applied minimum and maximum threshold values, which are logged in a ProcessingLog.txt file for traceability.

### Image outputs

**Fig. 3:**
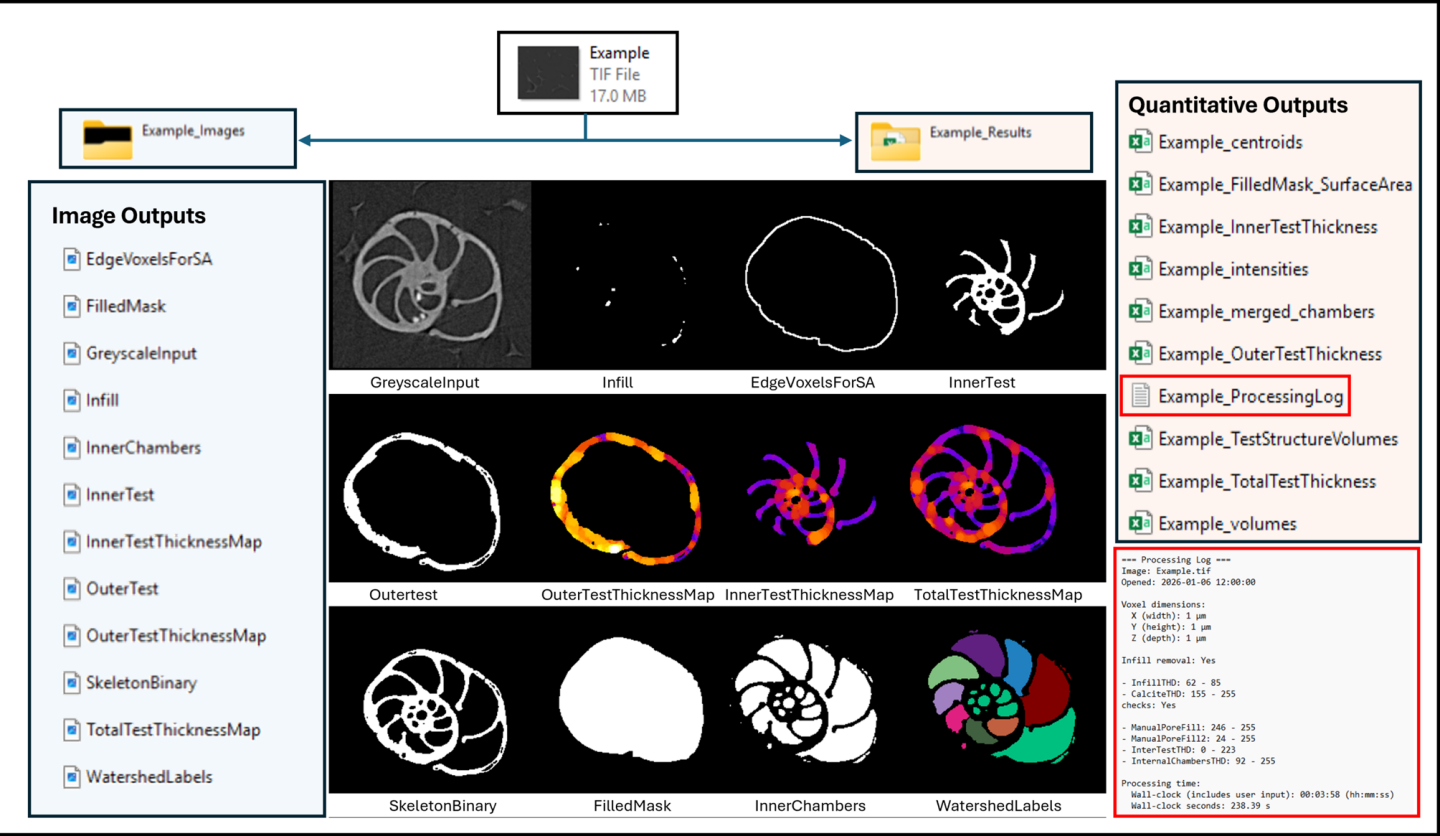
ForamJ output format. Processing in ForamJ generates a series of image outputs (listed left, shown centre), quantitative outputs (right, listed) and a log output (right, lower).

For each processed image stack, the macro generates a set of standardised image outputs, saved in the Sample_Images directory (where *Sample* refers to the base filename of the input image). These include both intermediate and final masks used in morphological and volumetric analyses summarised in Figure 3. The core outputs are:

- **Raw.tif** – the greyscale input image (post-infill removal, if applied).
- **SkeletonBinary.tif** – a binary mask representing the thresholded skeletal test.
- **FilledMask.tif** – a binary mask of the test after morphological closure.
- **InnerChambers.tif** – internal chamber space, derived by subtracting the skeletal mask from the filled test.
- **InnerTest.tif** – a binary mask of the internal septal region.
- **InnerTestThicknessMap.tif** – 3D thickness map of the inner test.
- **OuterTest.tif** – a binary mask of the outer test (test wall).
- **OuterTestThicknessMap.tif** – 3D thickness map of the outer test.
- **EdgeVoxelsForSA.tif** – binary edge mask used for surface area estimation.
- **WatershedLabels.tif** – segmented chamber stack, generated via 3D watershed.

If infill removal is enabled, an additional image (Infill.tif) is saved, containing the user-defined infill mask. Additional supporting images include DistanceMap.tif (Euclidean Distance Transform), SeedImage.tif (thresholded seed regions), and LabeledSeeds.tif (connected components of seed points), all used in chamber segmentation. All outputs are exported in .tif format using a consistent naming scheme for traceability.

### Quantification outputs

The macro generates several .csv files summarising morphometric and structural properties, saved in the Sample_Results directory. These include:

- **Sample_StructureVolumes.csv**: Total volumes (in voxels and µm^3^) for three key structures:
- SkeletonBinary: Representing the calcite shell as a binary image.
- FilledMask: The total test volume, with inner chambers filled.
- InnerChambers: Internal chamber space: Volume is calculated
  by counting binary voxels and scaling by the user-defined voxel volume.
- **Sample_FilledMask_SurfaceArea.csv**: An estimate of surface area is computed by detecting edge voxels in the *FilledMask* volume and multiplying the total edge voxel count by the average voxel face area (accounting for anisotropic voxel dimensions).
- **Sample_OuterTestThickness.csv and Sample_InnerTestThickness.csv**: Mean and distributional thickness metrics for both outer and inner test regions, computed using the BoneJ particle analysis module.
- **Sample_ChamberVolumes.csv, Sample_ChamberCentroids.csv, and Sample_ChamberIntensities.csv, combined into a CombinedChamberDetail.csv,** acting as a series of quantitative outputs derived from the *WatershedLabels* image. These include: Individual chamber volumes, 3D centroid coordinates and Mean voxel intensities (using *WatershedLabels* as both object and signal image).

At each manual segmentation step, users are prompted to input the minimum and maximum threshold values used. These values, along with voxel dimensions and key processing decisions (e.g. all threshold values that are used), are saved in a plain-text log file:

- **Sample_ProcessingLog.txt.** This log ensures transparency and supports reproducibility across datasets. The time taken to process the sample is also logged (Fig. 3).

### Statistical analysis

ForamJ is primarily an image processing tool rather than a statistical tool and as such statistical analyses must be determined by the user. For this showcase, the quantification of single samples for comparisons of benthic foraminifera (application 1) is reported in text as an absolute value per metric unless stated. Meanwhile, the reporting of 2 samples per species of planktonic foraminifera (application 2) was shown by the reporting of both values, unless stated. All graphs were made in GraphPad Prism (ver 10.4.1).

#### Application 1 study of benthic foraminifera

Seven samples (Table 2) were scanned at the University of Southampton, muvis X-Ray imaging centre. Benthic foraminifera recovered from deep-sea core sites can exhibit varying degrees of shell dissolution due to exposure to chemically corrosive bottom waters and porewaters following burial^43^. In contrast, clay-rich lithologies from continental shelf environments often yield exceptionally well-preserved foraminiferal specimens with glassy or translucent shell textures that are well suited for μCT image analysis^44^. Fossil benthic foraminiferal species *Cibicidoides alleni*^45^ and *Cibicidoides succedens*^46^ were selected from Eocene age sediments (~56 million years ago) obtained from the Ocean Drilling Program (ODP) Leg 174AX Bass River core, located in present-day New Jersey, USA^47^. The paleo-environment of Bass River makes it a preferred location for image analysis because it was a continental shelf setting, above the depths of potentially chemical corrosive deep waters while containing high clay content that acts as an impermeable, limiting fluid interaction and post depositional dissolution. A range of benthic foraminiferal sizes were selected for assessment to maximize the ranges in parameters (Table 1). While the *Cibicidoides* benthic foraminiferal shells are well-preserved chemical cleaning was conducted to minimise potential surficial contamination prior to μCT scanning . Whole marine sediment samples were disaggregated in Milli-Q water (18.2 Ω) using an orbital shaker and wet sieved to separate the coarse sand fraction > 63 µm for microfossil selection. Benthic foraminifera specimens were analysed from a narrow range of multiple size fractions 180–212, 212–250 and 250–300 µm and among sample depths spanning the entire PETM section to maximise the potential morphological variability in the sample set. Individual benthic foraminifera were placed in the centre of a watch glass under a stereomicroscope to be chemical cleaned prior to µCT analysis. The chemical cleaning protocol included sequential ethanol and Milli-Q water (18.2 Ω) rinses (3-5 times each) to remove adhered clay and sediments from the outside surface of the foraminiferal shell. Due to the variable solution viscosities, both ethanol and Milli-Q are utilized during cleaning to better facilitate surficial particle removal from the porous foraminiferal wall texture. Cleaned individuals were mounted into straws between evenly spaced layers of varying porosity foam to aid in distinguishing samples during scanning, as described^42^.

µCT scanning was completed in collaboration with muvis x-ray imaging centre, using a Zeiss Versa 610 x-Ray imaging system. Once mounted, high-resolution µCT scans were acquired using 110 kV, 10 W, with 1001 projections collected over 360°. An exposure time of 1.3s was used with a low-energy x-ray filter, yielding a total scan duration of 53 minutes per sample, 2x binning was used to provide a voxel size of 1.75 µm.

**Table 2:**
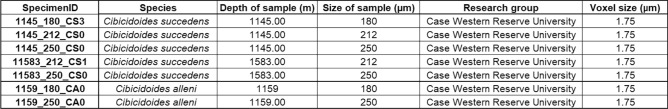
Benthic foraminifera sample information.

**Fig. 4:**
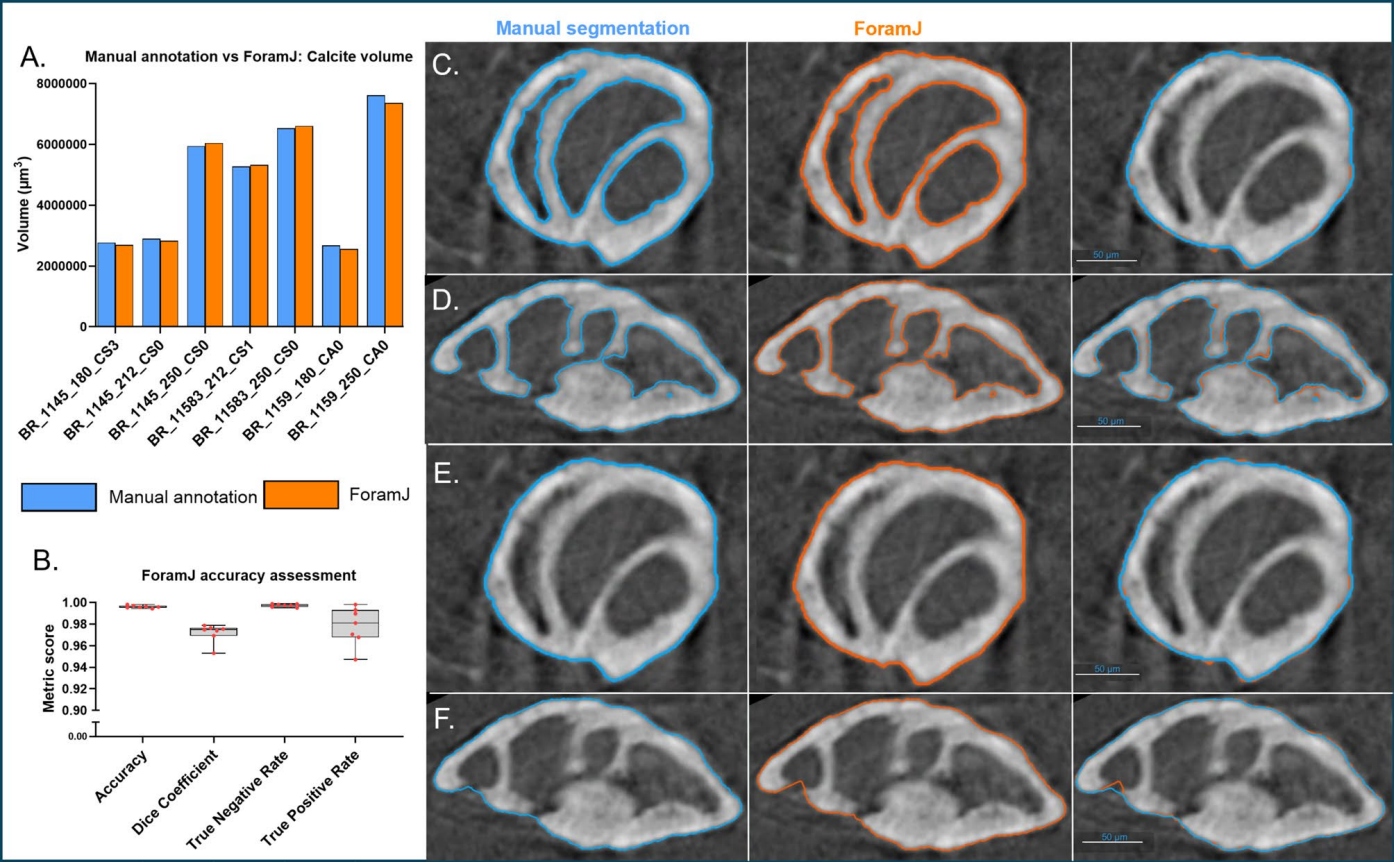
ForamJ validation. Processing of benthic foraminifera tests in Dragonfly (blue) and ForamJ (orange) for the comparative segmentation of calcite volume (**A**), and other comparative metrics, including accuracy, Dice score, true negative rate and true positive rate (**B**). (**C**-**F**) Show the comparative segmentation from the two methods, with the left hand column showing the manual segmentation result, right hand panel showing the ForamJ result (**C**) and (**D**) show this segmentation of the calcite volume, while (**E**) and (**F**) show the segmentation of the test volume.

For validation of the segmentation achieved by ForamJ, authors completed a similar workflow in Dragonfly 2024.1 relying on manual segmentation for the separation of infill, calcite and background. Authors chose to utilise the commercial software Dragonfly as the gold standard for segmentation and analysis. Utility of the 3D painter tool governed this, as a faster alternative to manual segmentation in Fiji. The segmentation for the calcite volume (Fig. 4A) was then compared as a volume versus the ForamJ output. Across all examined benthic foraminifera, there was a 1.30% difference in calcite volume between ForamJ and Dragonfly. Use of the segmentation comparison tool within Dragonfly facilitated the calculation of segmentation accuracy (0.9981 ± 0.001), dice score (0.97 ± 0.008), true negative rate (0.9972 ± 0.001), and true positive rate (0.9781 ± 0.016) suggestive that ForamJ reproduced manual segmentation faithfully (Fig. 4B). Comparison of manual segmentation and ForamJ-derived segmentation may be observed in Fig. 4 C-F.

**Fig. 5:**
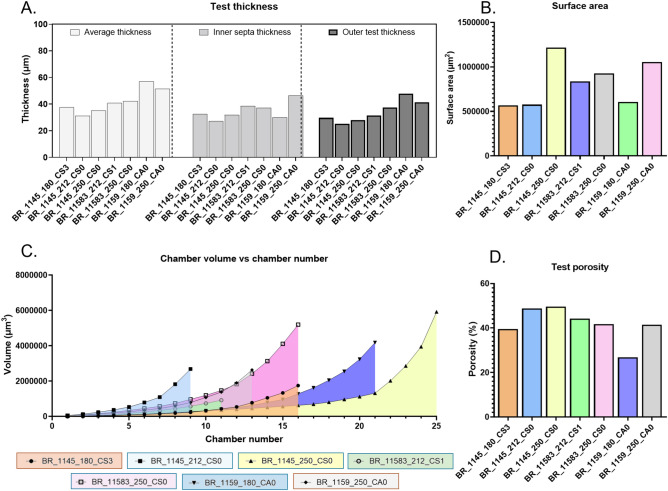
Quantitative morphometric measurements derived from µCT-based segmentation of benthic foraminiferal tests. ForamJ was used to segment µCT scans for the assessment of benthic foraminifer, yielding several morphometric measurements. (**A**) Test wall thickness measurements separated into average thickness (light grey), inner septa thickness (mid grey), and outer test thickness (dark grey) across multiple specimens. (**B**) Calculated test surface areas (µm^2^) for each specimen. (**C**) Chamber-by-chamber volumetric growth curves, showing the relationship between chamber number and chamber volume (µm^3^), with shaded regions highlighting specimen-specific trajectories. (**D**) Test porosity (%) calculated as the ratio of chamber volume to total test volume. Data is shown as mean values.

Subsequent morphometric analyses completed by ForamJ revealed clear inter-specimen variability in test architecture (Fig. 5). Calcite volumes ranged from 2.6×10⁶ μm^3^ (BR_1159_180_CA0) to over 6.6×10⁶ μm^3^ (BR_11583_250_CSO), broadly reflecting differences in test size. Test porosity spanned 26.7% – 49.7%, with most specimens clustering between 39–44%. Wall thickness ranged from 31.2 μm in BR_1145_212_CS0 to 56.9 μm in BR_1159_180_CA0, with inner septa consistently thinner than outer test walls. Test surface areas varied between ~ 5.7×10^5^ μm^2^ and > 1.2×10⁶ μm^2^, while chamber volumes produced distinct growth trajectories: BR_1145_250_CSO, with 25 chambers, exhibited the steepest cumulative increase in contrast to the smaller, more compact form of BR_1145_212_CS0 (9 chambers). Together, these measurements highlight marked differences in skeletal investment and chamber organisation across specimens.

#### Application 2: ForamJ for the study of planktonic foraminifera

To showcase the utility of ForamJ to the wider foraminifera community, two individuals from seven planktonic foraminifera species were processed using ForamJ, accessed from the repository provided by^30,^ summarised in Table 3 This application was used to showcase the variety of test morphologies that could be assessed using ForamJ. The μCT image collection of planktonic foraminifera compiled by Siccha et al.^30^ represents a unique benchmark dataset in the micropaleontological research community, providing an optimal resource for developing image processing techniques. This is primarily because it is open access and that datasets are supplied as raw greyscale files, without prior segmentation or processing, which is important due to foraminiferal μCT processing being laboratory-specific and lacking a standardised reference sample set or protocol. Moreover, the taxonomy of all planktonic foraminifera specimens has been independently validated and are curated, enabling follow-up investigations and repeated measurements when necessary. Collectively, for these factors make the dataset exceptionally well-suited for future inter-comparative studies, as it allows for consistent re-evaluation of μCT analyses.

**Table 3:**
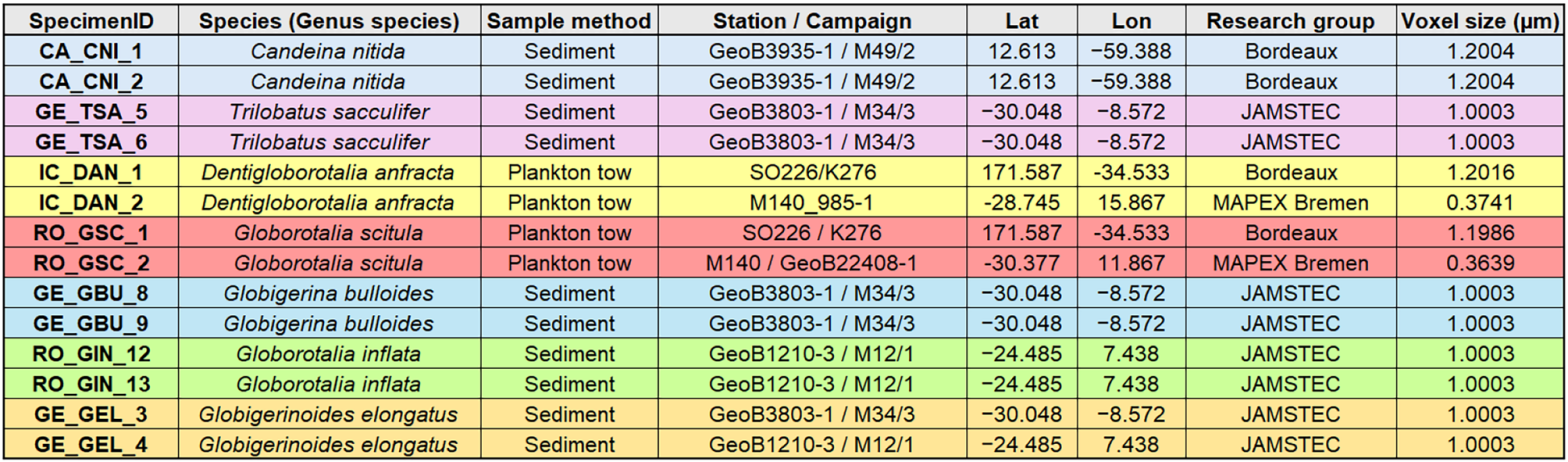
Planktonic foraminifera sample information.

Datasets utilised and shown in this table were accessed from the open repository provided by^30^.

**Fig. 6:**
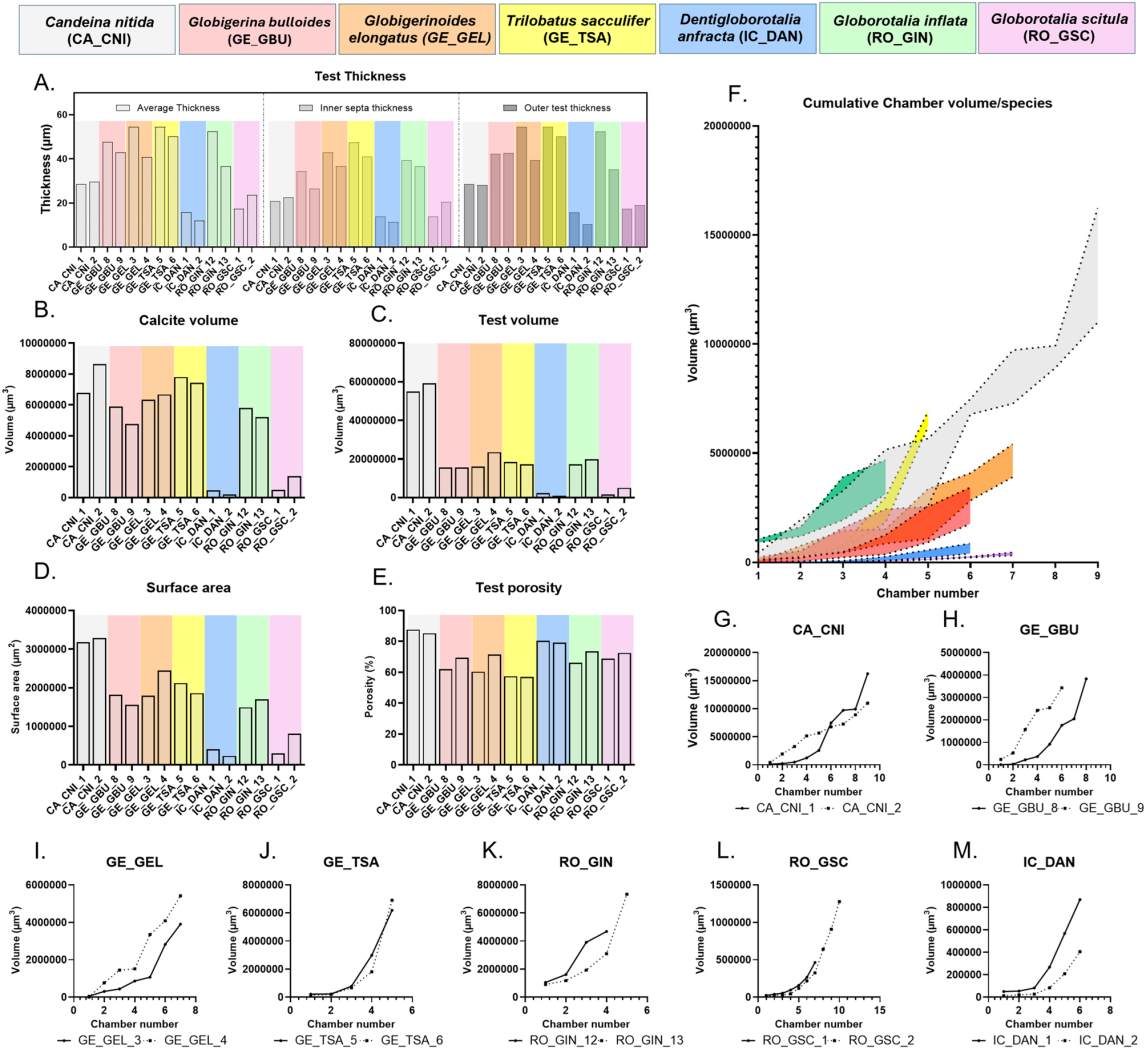
Inter-species comparison of test architecture in planktonic foraminifera using ForamJ. Quantitative morphometric traits were extracted from 3D reconstructions of multiple species to assess variation in test architecture for the assessment of multiple species of planktonic foraminifera: (**A**) Test thickness metrics separated into average, inner septa, and outer test thickness, (**B**) Calcite volume, (**C**) total test volume, (**C**) surface area and (**D**) porosity measured across species. (**F**) Cumulative chamber volume curves show species-level growth trajectories with data shown as mean ± SD per species. (**G**–**M**) Displays the chamber-by-chamber growth curves for individual species, highlighting similarities between individuals of the same species. Datasets utilised in this figure were accessed from the open repository provided by^30^.

µCT-derived morphometric analyses revealed pronounced interspecific differences in test architecture among the examined planktonic foraminifera (Fig. 6), including test thickness (Fig. 6A), calcite volume (Fig. 6B), test volume (Fig. 6C), surface area (Fig. 6D), test porosity (Fig. 6E), and chamber volume trajectories (Fig. 6F). Individual species chamber volume trajectories are also shown, displaying intraspecies variability (Fig. 6G-M).

*Candeina nitida* (CA_CNI) species displayed the highest porosities (85.4%, 87.7%), combined with the thinnest walls, with inner septal thicknesses of 12.0 µm and 12.9 µm and outer wall thicknesses of 28.5 µm and 29.7 µm. Despite their delicate structure, chamber volumes reached 4.8 × 10⁷ µm^3^ and 5.0 × 10⁷ µm^3^, supported by calcite volumes of 6.8 × 10⁶ µm^3^ and 8.6 × 10⁶ µm^3^. Growth trajectories were relatively linear, reflecting steady volumetric expansion (Fig. 6G). *Globigerina bulloides* (GE_GBU) species exhibited substantially thicker walls, with inner septa of 26.4 µm and 34.4 µm and outer walls of 42.3 µm and 42.7 µm, alongside lower porosities (62.1%, 69.5%). Individual chambers frequently exceeded 9.6 × 10⁶ µm^3^, contributing to total test volumes of 1.5 × 10⁸ µm^3^ and 1.6 × 10⁸ µm^3^, supported by calcite volumes of 4.7 × 10⁶ µm^3^ and 5.9 × 10⁶ µm^3^. Chamber growth was stepwise, with disproportionately large additions during the outer whorl (Fig. 6H). *Globigerinoides elongatus* (GE_GEL) species combined moderate porosities (57.4%, 71.7%) with inner septal thicknesses of 36.7 µm and 42.9 µm and outer wall thicknesses of 39.3 µm and 54.6 µm. Chamber volumes reached 1.6 × 10⁷ µm^3^, with calcite volumes of 6.3 × 10⁶ µm^3^ and 6.7 × 10⁶ µm^3^, and cumulative growth curves showed marked late-stage expansion (Fig. 6I). Similarly, *Trilobatus sacculifer* (GE_TSA) exhibited porosities of 56.9% and 57.4%, with inner septa of 40.1 µm and 47.5 µm and outer walls of 50.1 µm and 54.5 µm. Chamber volumes were 1.2 × 10⁷ µm^3^ and 1.4 × 10⁷ µm^3^, supported by calcite volumes of 7.1 × 10⁶ µm^3^ and 7.8 × 10⁶ µm^3^ (Fig. 6J).

*Globorotalia inflata* (RO_GIN) exhibited intermediate porosities (60.7%, 79.3%) with inner septa of 30.4 µm and 34.8 µm and outer walls of 32.5 µm and 36.7 µm. Chamber volumes reached 1.1 × 10⁷ µm^3^ and 1.4 × 10⁷ µm^3^, supported by calcite volumes of 5.2 × 10⁶ µm^3^ and 5.8 × 10⁶ µm^3^, with steady volumetric increases across successive chambers (Fig.6K). By contrast, *Globorotalia scitula* (RO_GSC) produced compact tests with porosities of 72.5% and 73.1%, inner septa of 17.3 µm and 21.3 µm, and outer walls of 25.7 µm and 36.2 µm. Chamber volumes were 3.6 × 10⁶ µm^3^ and 1.1 × 10⁷ µm^3^, supported by calcite volumes of 1.4 × 10⁶ µm^3^ and 5.0 × 10⁶ µm^3^. Surface areas were comparatively high (2.3 × 10⁶ µm^2^, 2.6 × 10⁶ µm^2^), reflecting greater external complexity despite reduced volumetric growth (Figure 6L). *Dentigloborotalia anfracta* (IC_DAN) showed higher variability, with porosities of 60.2% and 80.4%, inner septa of 29.3 µm and 36.7 µm, and outer walls of 25.7 µm and 36.2 µm. Chamber volumes were comparatively small (7.7 × 10⁶ µm^3^, 9.6 × 10⁶ µm^3^), and calcite volumes limited (2.0 × 10⁶ µm^3^, 4.6 × 10⁶ µm^3^), resulting in shallow cumulative growth profiles (Fig. 6M). Together, these data demonstrate a continuum of test morphologies, ranging from thin-walled, highly porous tests with rapid chamber proliferation *Candeina nitida* (CA_CNI) to thick-walled, low-porosity taxa with thicker test architectures consistent with a greater proportion of gametogenic calcite^48^. *Trilobatus sacculifer* (GE_GBU), with other species occupying intermediate positions defined by moderate porosity, chamber expansion, and wall thickness.

### Throughput and portability

**Fig. 7:**
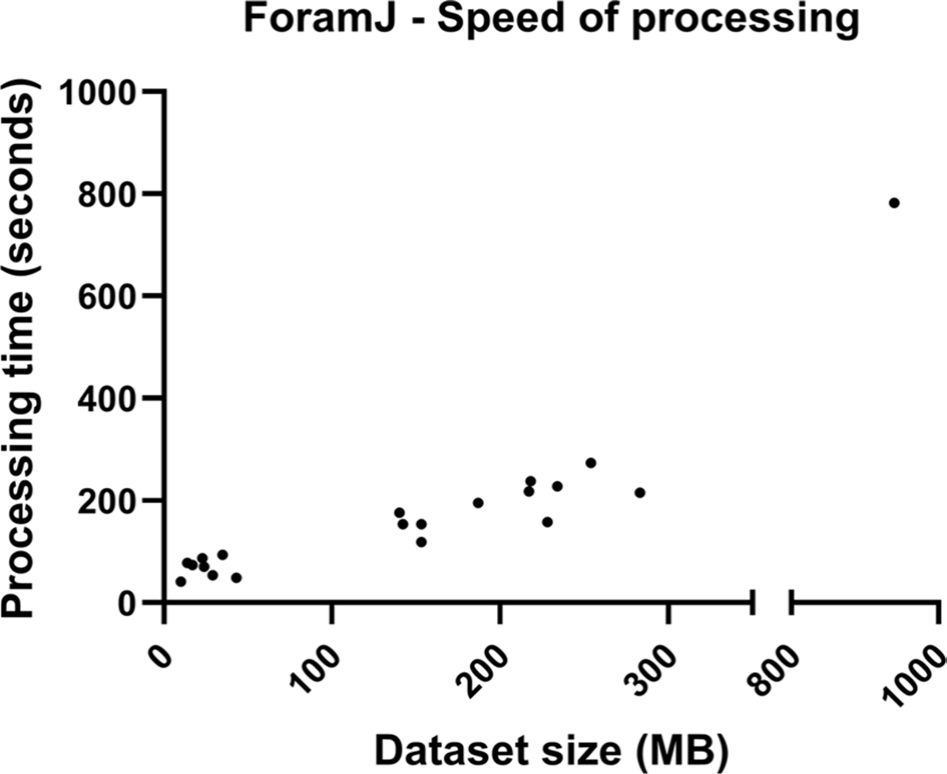
ForamJ processing speed. Dataset size (MB) is plotted against processing time (seconds), showcasing the scalable relationship even on low-capacity workstations.

All processing on applications 1 and 2 were completed on a non-specialist laptop, equipped with 8GB of RAM. Datasets used varied across two orders of magnitude; with the speed of processing varying significantly as a function of dataset size (Fig. 7). The smallest dataset (RO_GSC_1) was 14 MB and took 78 seconds to process, while the largest dataset (RO_GSC_2) was 940 MB and took 782 seconds to process. The median dataset size was 142 MB, with a processing speed of 154 seconds. Processing of these datasets on a relatively low-capacity workstation highlights ForamJ’s portability even for the processing of larger (~1 GB) datasets.

## Discussion

ForamJ provides several advantages over existing software; it provides a number of metrics that are defined by the need within the existing literature. It does so in open source, non-commercial software, allowing for a one-stop user experience for the analysis of µCT datasets of foraminifera, with applications to both benthic and planktonic foraminifera.

Importantly, it addresses a number of limitations of existing software. Namely, it is contained within the open-source environment of Fiji-ImageJ rather than relying on specialist, often costly, commercial software. ForamJ meanwhile provides a user-friendly user walkthrough of processing alongside an easy, lightweight setup, allowing non-specialist users to complete morphological analyses.

The workflow also acts to improve on throughput, by providing an optional “Single” or “Batch” mode. This “Single” mode allows for the user to test and validate the workflow versus existing methods, while the batch mode allows the user to move through high sample numbers. Additionally, the uniform export of .tiff stacks and .csv files allows for the user to combine ForamJ with their own downstream analysis pipelines.

ForamJ also attempts to circumvent the potential for non-homogenous µCT scans, should greyscale values vary between or within sessions or scanners. The use of a manual thresholding step allows the user to control this element of segmentation, without requiring a complex segmentation approach, utilising complex workflows, machine learning or even deep learning algorithms. While this approach has also been used to reduce infill contamination, it is noted that this is primarily designed for the removal of discrete infilling (i.e. siliciclastic), and that more complex infill removal may be facilitated by dedicated segmentation plugins such as WEKA^49^ or more advanced cleaning protocols prior to imaging. Additionally, while integration within the open-source Fiji environment provides a number of advantages, allowing for the prioritisation of throughput, quantification and reproducibility, it does possess limited 3D rendering versus commercial software such as ORS Dragonfly, Aviso/Amira, VG Studio and IPSDK. Currently, ForamJ does not prioritise this 3D rendering, although opening of an image stack outputted by ForamJ, and rendering using the 3D Viewer plugin, does allow for this need to be met in some capacity and improved rendering capabilities does provide an exciting angle for future releases.

Similarly, the desire to isolate pores within the test itself is a need that is currently unmet by ForamJ. Pores within the test are often poorly resolved and will require the development of further segmentation-focused modules, which the authors anticipate being added to ForamJ in future updates and releases. On this note, users may request and provide feedback to ForamJ using the email address ForamJDevs@gmail.com. Finally, while ForamJ is provided as a freely available software, the responsibility of validating individual results lies with the user, not the developer.

## Supplementary Information

Supplementary Information 1

Supplementary Information 2

Supplementary Information 3

Supplementary Information 4

Supplementary Information 5

Supplementary Information 6

Supplementary Information 7

Supplementary Information 8

## Data Availability

Datasets showcased for applications (1) and (2) are publicly available. For application (1), data is accessible in the supplementary material of this article, while (2) is freely available by^30^, using the following URL: 10.1594/PANGAEA.949585.
