# Supplementary figures and images for: ForamJ – A tool for the reproducible, semi-automated analysis of foraminifera micro computed tomography datasets
Source: Sci Rep. 2026 Mar 24;16:14818. doi: 10.1038/s41598-026-43276-3 (PMC13168569; doi:10.1038/s41598-026-43276-3)

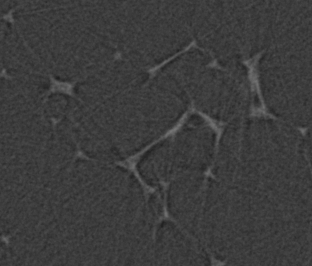

Supplement: Supplementary file 1 — Supplementary Information 1 [file 41598_2026_43276_MOESM1_ESM.tif]

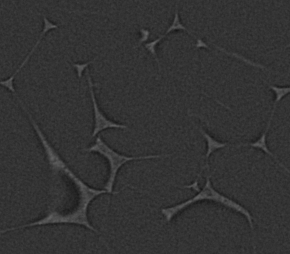

Supplement: Supplementary file 2 — Supplementary Information 2 [file 41598_2026_43276_MOESM2_ESM.tif]

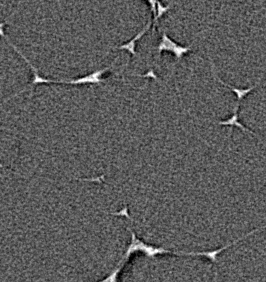

Supplement: Supplementary file 3 — Supplementary Information 3 [file 41598_2026_43276_MOESM3_ESM.tif]

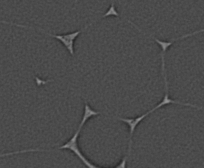

Supplement: Supplementary file 4 — Supplementary Information 4 [file 41598_2026_43276_MOESM4_ESM.tif]

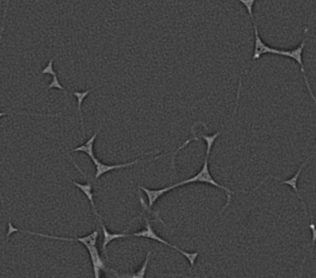

Supplement: Supplementary file 5 — Supplementary Information 5 [file 41598_2026_43276_MOESM5_ESM.tif]

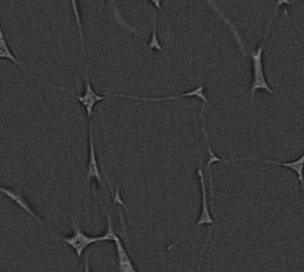

Supplement: Supplementary file 6 — Supplementary Information 6 [file 41598_2026_43276_MOESM6_ESM.tif]

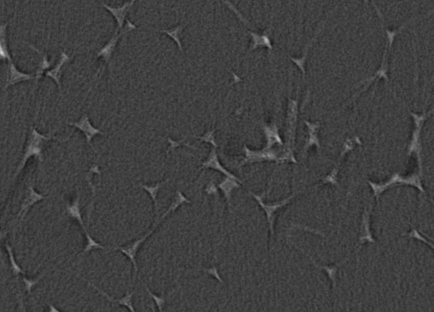

Supplement: Supplementary file 7 — Supplementary Information 7 [file 41598_2026_43276_MOESM7_ESM.tif]
